# Ötzi the Iceman: forensic 3D reconstructions of a 5300-year-ago murder case

**DOI:** 10.1007/s00414-025-03510-5

**Published:** 2025-05-21

**Authors:** Chiara Villa, Sara Larsen, Albert Zink, Niels Lynnerup

**Affiliations:** 1 Department of Forensic Medicine, University of Copenhagen, Copenhagen, Denmark; 2 Institute for Mummy Studies, EURAC, Bolzano, Italy

**Keywords:** PMCT, Animation, 3D models, Hematoma

## Abstract

The cause of death of Ötzi the Iceman has been the subject of extensive investigation. A deep wound on his left shoulder and an arrowhead lodged between his rib cage and left scapula have been central to determining his final moments. While initial CT analyses concluded that exsanguination due to a lacerated left subclavian artery was the cause of death, recent research has suggested that the injury might not have been immediately fatal. This study re-analyzed the 2013 CT scans using a forensic approach to assess the shoulder injury in greater detail, by creating 3D models of the affected anatomical structures and calculating relevant tissue volumes. Additionally, forensic animation techniques were applied to reconstruct Ötzi’s likely posture at the moment of impact. A previously unidentified hematoma with an approximate volume of 110 mL was observed, suggesting that death from blood loss alone may not have been immediate, although external hemorrhage cannot be excluded. Furthermore, forensic animation demonstrated that a straight trajectory of the arrow aligns with both the scapular lesion and arterial laceration, providing new insights into the injury mechanism. These results underscore the value of 3D segmentation and modeling in forensic pathology, offering enhanced reconstructions of traumatic events in both contemporary and archaeological contexts.

**Supplementary Information:**

The online version contains supplementary material available at 10.1007/s00414-025-03510-5.

## Introduction

Over the past two decades, forensic medicine has embraced advanced imaging techniques, including CT and MR scanning, as valuable complements to traditional autopsy. These imaging modalities have progressed beyond their initial role as basic visualization tools and are now capable of enabling advanced analyses, such as virtual body animations [1–6].

3D models produced through these imaging modalities are highly detailed and tailored to each individual case, accurately reflecting the victim’s proportions and the precise locations of injuries. Furthermore, these 3D models can be animated to simulate likely body positions and movements at the time of an incident [7–11].

CT scanning is an essential tool for studying mummies, and, as in forensic medicine, it enables forensic reconstruction to be performed in ancient cases. Iceman, also known as Ötzi, is one of the oldest known murder cases in human history and *“the most intensely investigated human corpse in the history of the scientific study of mummies”*, as noted by Zink and Maixner (2019). Imaging analyses—including X-ray, CT —along with biomolecular analyses of DNA, proteins, and lipids, as well as palaeobotanical investigations, have all contributed to our understanding of such ancient remains [12].

On September 19, 1991, the mummified body of a man was discovered in the Ötztal Alps at an altitude of 3,210 m, on the border between Austria and Italy. This glacier mummy, later named the Iceman, was determined to date back to approximately 3300 B.C, based on radiocarbon dating [13]. Scientific analysis revealed that the Iceman was around 40–50 years old at the time of his death, with a height of approximately 160 cm and an estimated weight of 61 kg; his remains showed signs of degenerative changes in several joints, including the hips, shoulders, knees, and spine, along with evidence of arteriosclerosis and gastrointestinal problems; he had also suffered healed fractures to his ribs (see for example [12–28]).

From a forensic pathological perspective, particular attention has been given to a deep wound on his left shoulder and an arrowhead lodged between his rib cage and left scapula. Investigations using X-ray imaging and CT scans concluded that the cause of death was exsanguination due to a puncture wound inflicted by an arrowhead, which had lacerated the left subclavian artery [29–32]. However, a recent study [33] suggested that the injury might not have been immediately fatal, and the Iceman could have survived for several hours after being wounded.

The aim of this study was to re-analyze the 2013 CT scans and assess the shoulder injury by applying a forensic approach. 3D models focusing specifically on the shoulder structures were created and volume of the relevant structure was calculated. Finally, by applying forensic animation techniques, this study seeks to determine the trajectory of the arrow, reconstruct the likely posture of the Iceman at the moment of shooting.

## Materials and methods

### CT scanning

The CT images used in this study were performed in 2013 at Bolzano Hospital, using a Siemens Somatom Definition Flash with the CT parameter as reported in Table 1.

**Table 1:** CT parameters of the different imaging sessions

	Whole-body scanning	Head and thorax scanning	Pelvis and legs scanning
kV	120	120	120
mAs	300	350	300
Field of view	500 mm	500 mm	500 mm
Table Feed per rotation	23	7.2	23
Slice thickness	1.5 mm	1.5 mm	1.5 mm
Pitch	0.6	0.6	0.6
Slice increment	1	1	1
Reconstruction algorithm	B31s	B70s	B31s
Pixel size	0.976	0.976	0.976

The entire body was scanned in different sessions due to the complicated posture: whole-body CT from head to feet, excluding the left leg and part of the left trunk; head to upper abdomen; and lower extremities from the pelvis to the feet (Supplemental Fig. 1). The right hand was not scanned.

### 3D segmentations and volumes

3D models of the skeleton, relevant internal organs, and arrow paths were created using Mimics software v. 24 [34]. The 3D models of the skin, bones, blood vessels, hematoma, and arrowhead were generated through manual segmentation, with an initial Hounsfield unit (HU) range from − 700 to 3071. A whole-body 3D model was constructed by combining various 3D models generated from the different CT series. This 3D model was then used in the animation process. Volumes of the brain, cranial cavity, and hematoma were extracted using the software’s automatic tools after creating the 3D models. The brain shrinkage, based on the endocranial volume, was used as a proxy to estimate dehydration and the extent of the hematoma. We applied a similar inverse calculation that has been previously applied to fossils, dry skulls, and mummies [35–39].

### 3D animation and forensic reconstructions

The animation process was carried out using 3ds Max, as described by Villa et al. [11], utilizing a fully rigged 3D human anatomy model [40]. Since Iceman’s body was found to be anterior-posteriorly compressed (Supplemental Fig. 2), we used the skeleton of a recently deceased individual with similar body proportions as a reference to repositioning Iceman’s bones into an anatomical posture. The CT scan of the recent deceased was randomly selected from the database of the Department of Forensic Medicine. The criteria were similar body height and shoulder breadth to those of Iceman. 3D models of the bones were generated through automatic segmentation, applying a HU range from 150 to 3071.

The 3D models of Iceman’s body and those of the recent deceased were aligned using the shoulder and pelvis as reference points (Supplemental Fig. 3). Subsequently, each bone in the 3D human anatomy model was adjusted in size and rotation to match the skeletal structure of both Iceman and the recently deceased individual as closely as possible. For a detailed description of all the steps, please refer to Villa et al. [11].

## Results

Here, we provide a forensic pathological description of the lesion as observed through CT scanning and 3D visualizations of isolated structures highlighting the most significant findings. Much of the available information has already been reported in previous publications [27–30]. For the first time, we present new findings, including a larger extent of the hematoma, with precise 3D segmentation and volume calculation provided. Additionally, we offer accurate measurements of the bone perforation and wound channel. 3D animation of possible posture at the time of the shooting was created.

### Description of the lesion and 3D visualizations from CT scanning

**Fig. 1:**
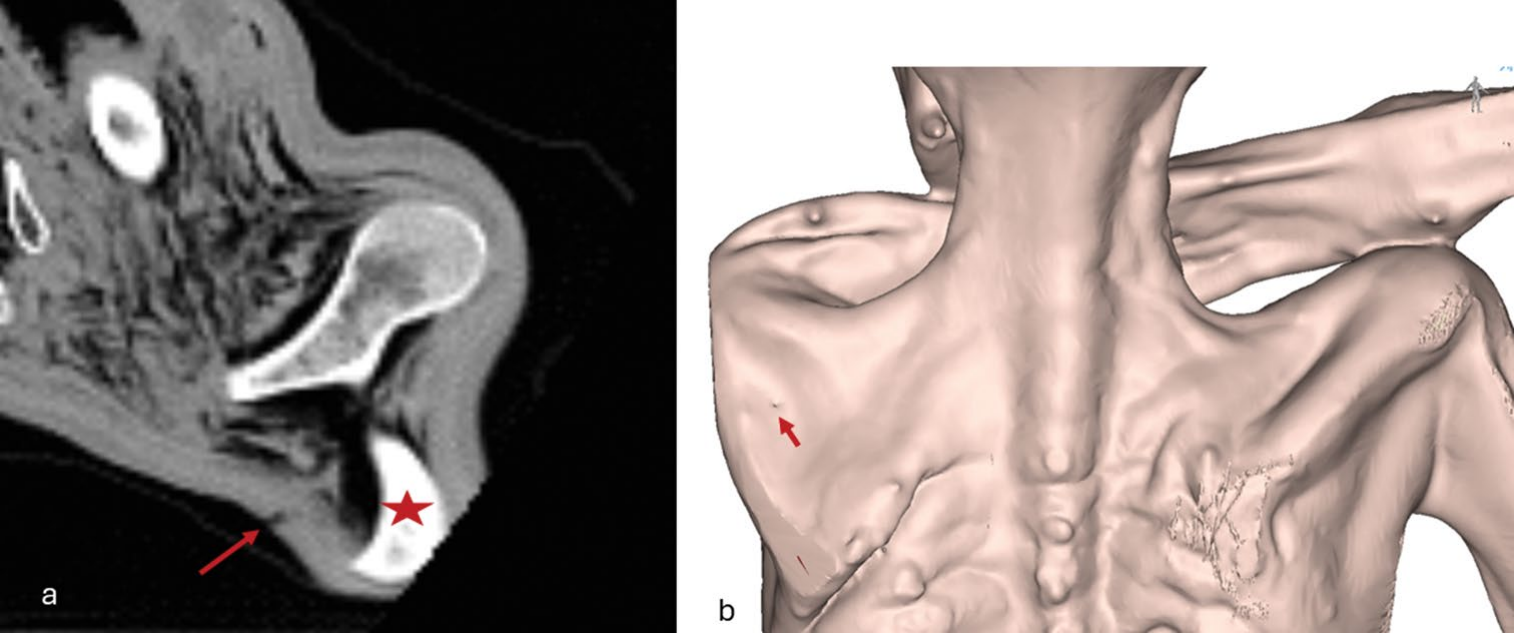
Visualization of the puncture wound: (**a**) Axial image showing the injury at the skin level, indicated by the arrow; the star marks the left scapula. (**b**) 3D visualization of the skin, showing the injury indicated by the arrow

**Fig. 2:**
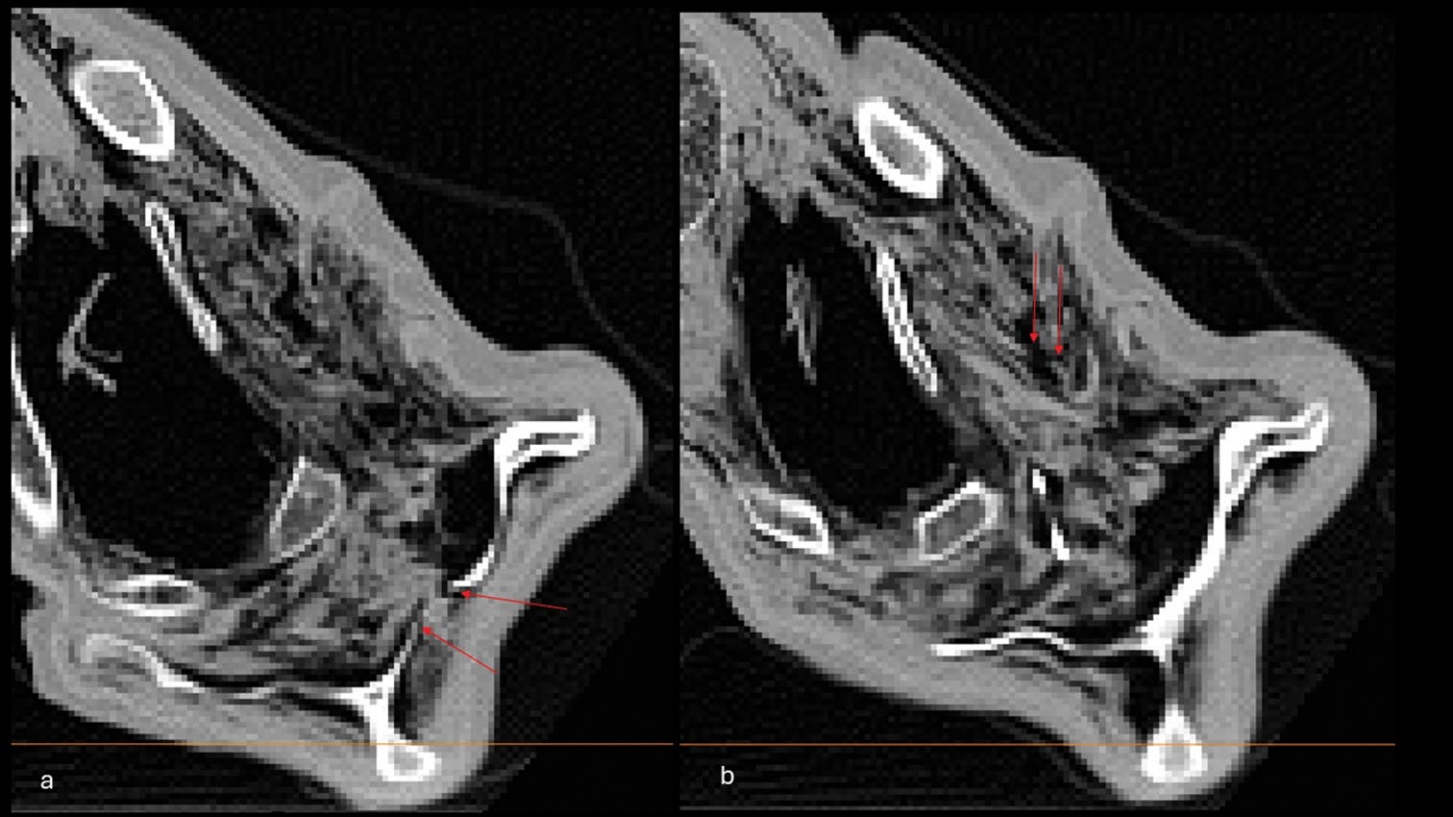
Axial images showing (**a**) the lesion in the scapular fossa and (**b**) the laceration of the subclavian artery

**Fig. 3:**
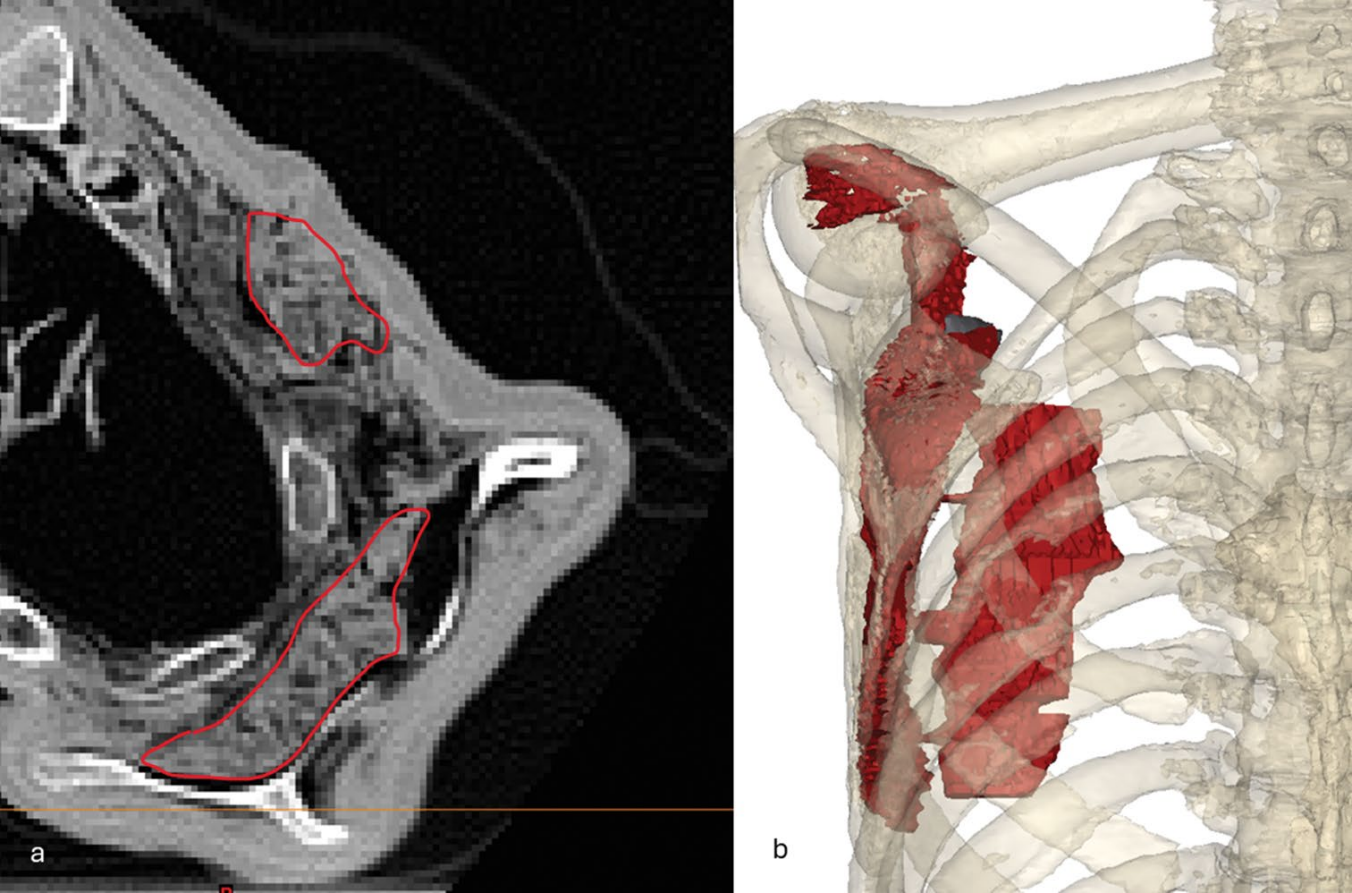
Visualizations of the hematoma: (**a**) axial image indicating the two areas where the hematoma was identified. (**b**) 3D visualization of the identified hematoma

The body of Iceman appears dehydrated, shrunken, and compressed frontally and dorsally. As seen and measured on the CT scans, a puncture wound approximately 0.3 cm in diameter is observed in the left shoulder region in the upper left side of the back (Fig. 1); as measured on the whole-body 3D model, the lesion is situated 12 cm lateral of the spine and 128 cm above the heel of the right foot. CT scans reveal damage to the deltoid muscle, the infraspinatus and subscapularis muscles, the scapular fossa, and a laceration of the subclavian artery, as previously reported (Fig. 2). A hematoma is identified in the area between the scapula and the ribcage, extending anteriorly into the upper thoracic region and further downward along the anterior thoracic wall for a total length of 9.3 cm– as measured on the 3D model (Fig. 3).

**Fig. 4:**
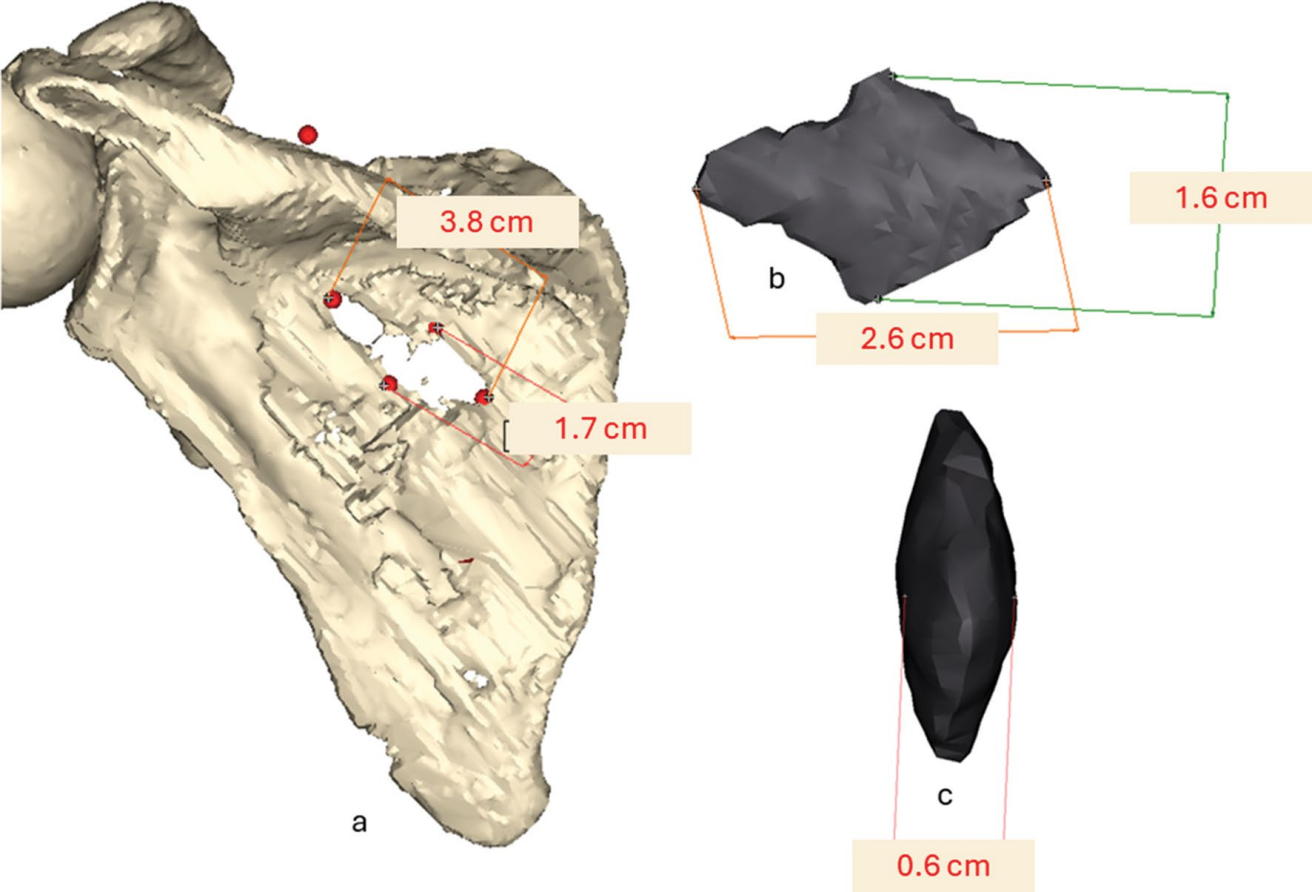
3D visualizations: (**a**) The left scapula with the dimensions of the bone defect. (**b**) Lateral view of the arrowhead with its dimensions. (**c**) Frontal view showing the width measurement of the arrowhead

The scapular fracture involves the supraspinous and infraspinous fossae, forming a rectangular osseus defect measuring 3.8 × 1.7 cm– as measured on the 3D model (Fig. 4a). A foreign body, identified as an arrowhead, is lodged between the ribs and scapula. The arrowhead measures 2.6 × 1.6 cm with a thickness of 0.6 cm– as measured on the 3D model (Fig. 4b and c).

**Fig. 5:**
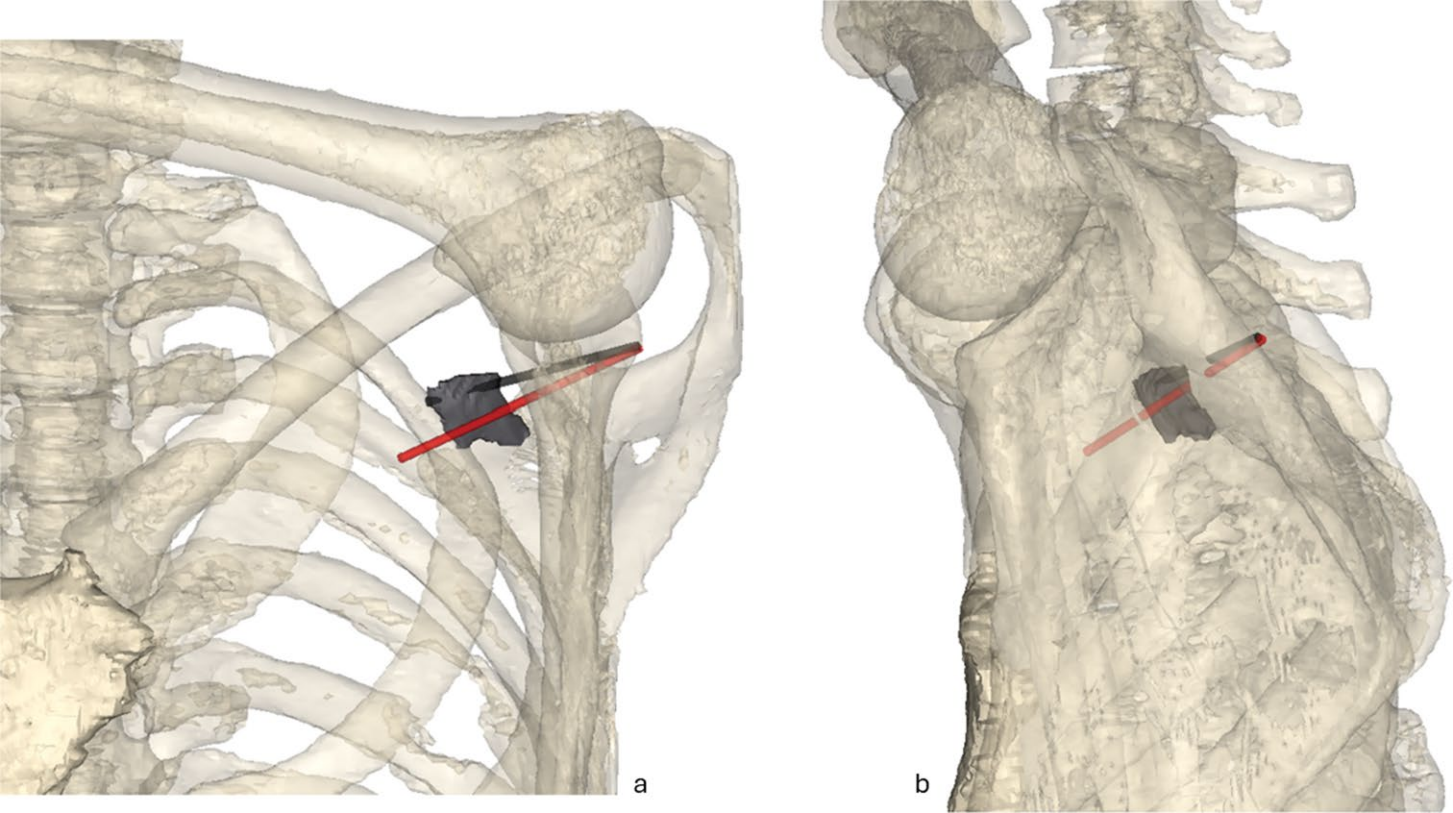
3D visualizations of the wound canals. The wound canal from the skin injury to the subclavian artery laceration is shown in red, while the wound canal from the skin injury to the arrow tip is shown in black. (**a**) Frontal view. (**b**) Lateral view

Two wound canals were traced from the skin lesion to the arrow tip and to the subclavian artery laceration. They measure 5 and 6 cm, respectively (as measured on the CT images), with a similar trajectory oriented forward to the right and slightly downward (Fig. 5) However, determining the precise direction and length of the wound canal is hindered by the current position of the body, with the arm positioned forward, altering the alignment of the scapula. In its present orientation, the lesions of the skin, scapula, and subclavian artery do not align in a straight line.

### Hematoma volume

The total volume of the hematoma in the shoulder area was determined to be 64 cm³, corresponding to a corrected volume of approximately 105 cm³, equivalent to roughly 110 mL of blood. The corrected volume was calculated based on an estimated 61% reduction in tissue volume due to dehydration. This reduction was derived from 3D models of the brain and cranial cavity (Supplemental Fig. 4). The cranial cavity had an approximate volume of 1574 cm³, while the brain volume measured 626 cm³, indicating a 61% reduction in brain volume.

### 3D animations

**Fig. 6:**
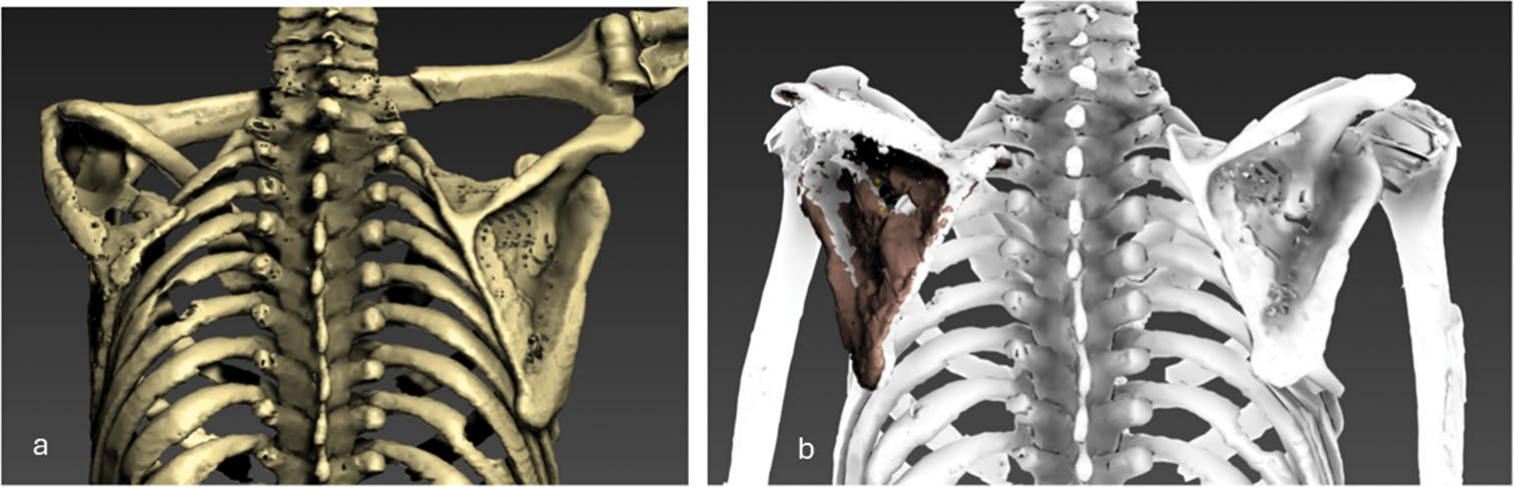
3D visualizations of the thorax seen from the back. (**a**) In its original position. (**b**) after the animation

**Fig. 7:**
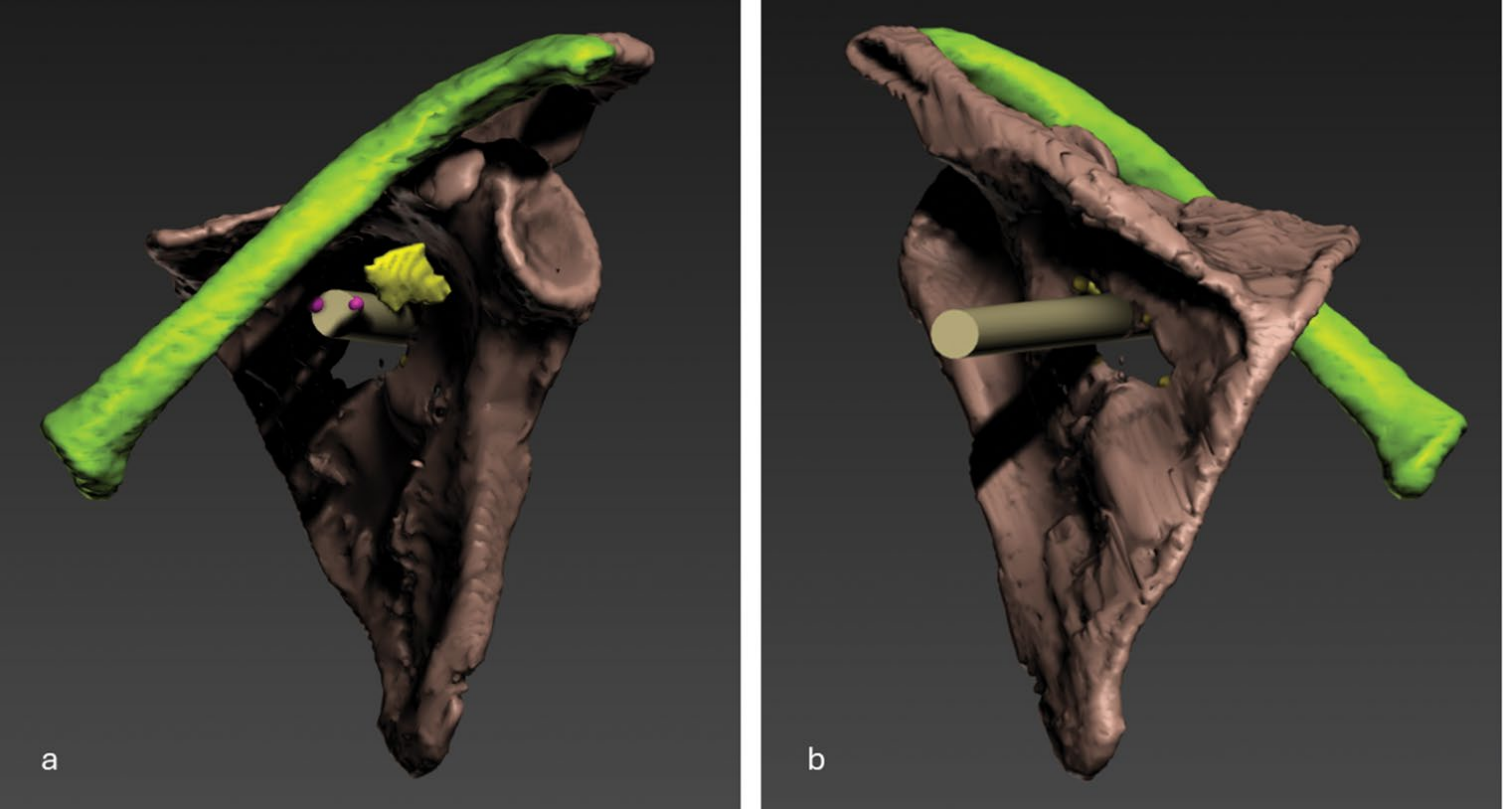
3D visualizations after the animation, showing the clavicle (green), scapula (brown), arrowhead (yellow), arterial laceration (pink), and wound canal (light brown). (**a**) Frontal view; (**b**) back view

As mentioned earlier, in its current orientation, the lesions of the skin, scapula, and subclavian artery do not align in a straight line. Using animation techniques, we repositioned Iceman’s arm along the body, thereby adjusting the alignment of the bones in the left shoulder (Fig. 6). Upon closer examination of the lesion, it became evident that the wound canal from the skin to the arterial laceration follows a forward and straight trajectory (Fig. 7).

**Fig. 8:**
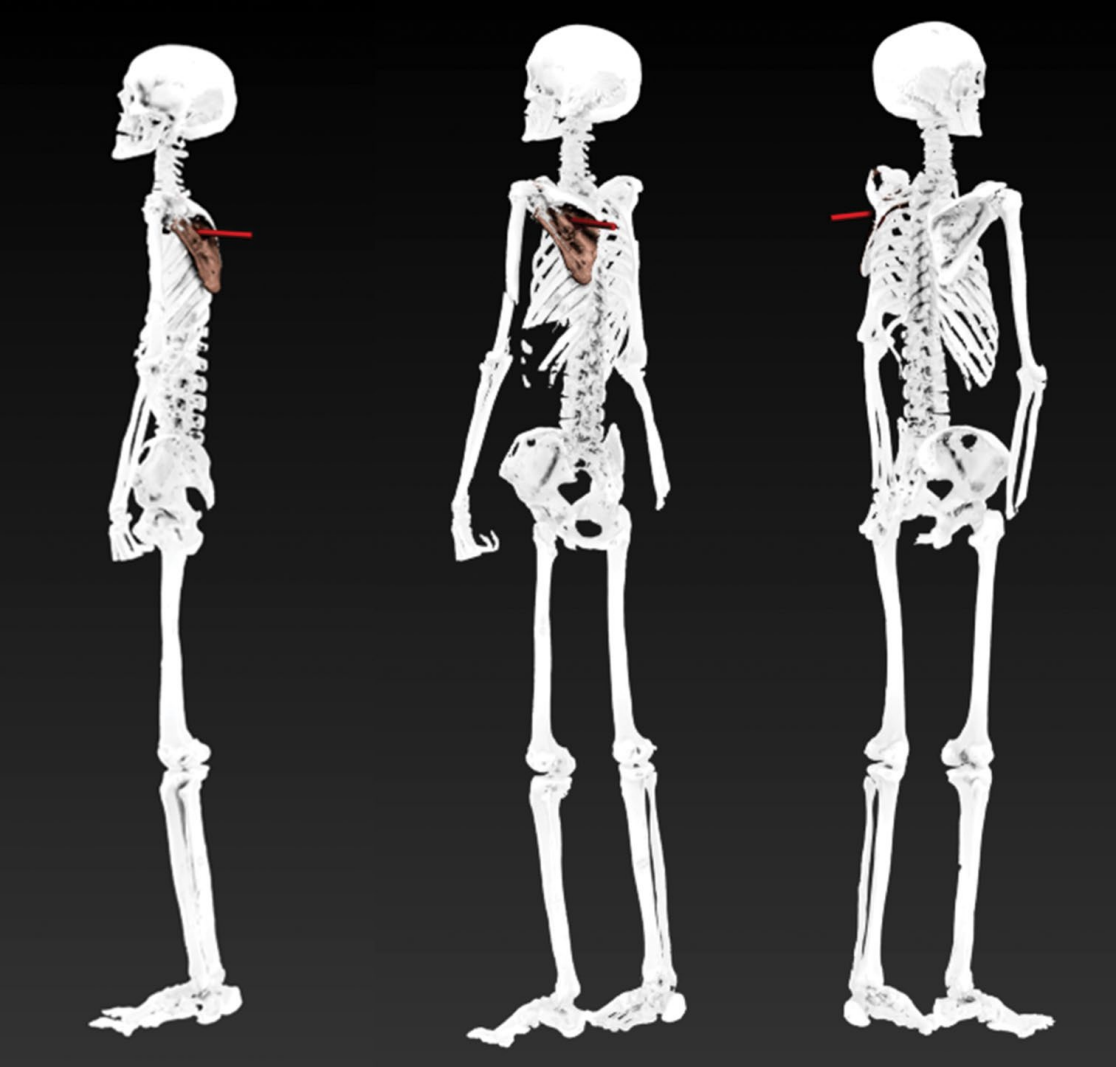
A straight posture of Iceman at the time of the shooting, seen from different angulations

**Fig. 9:**
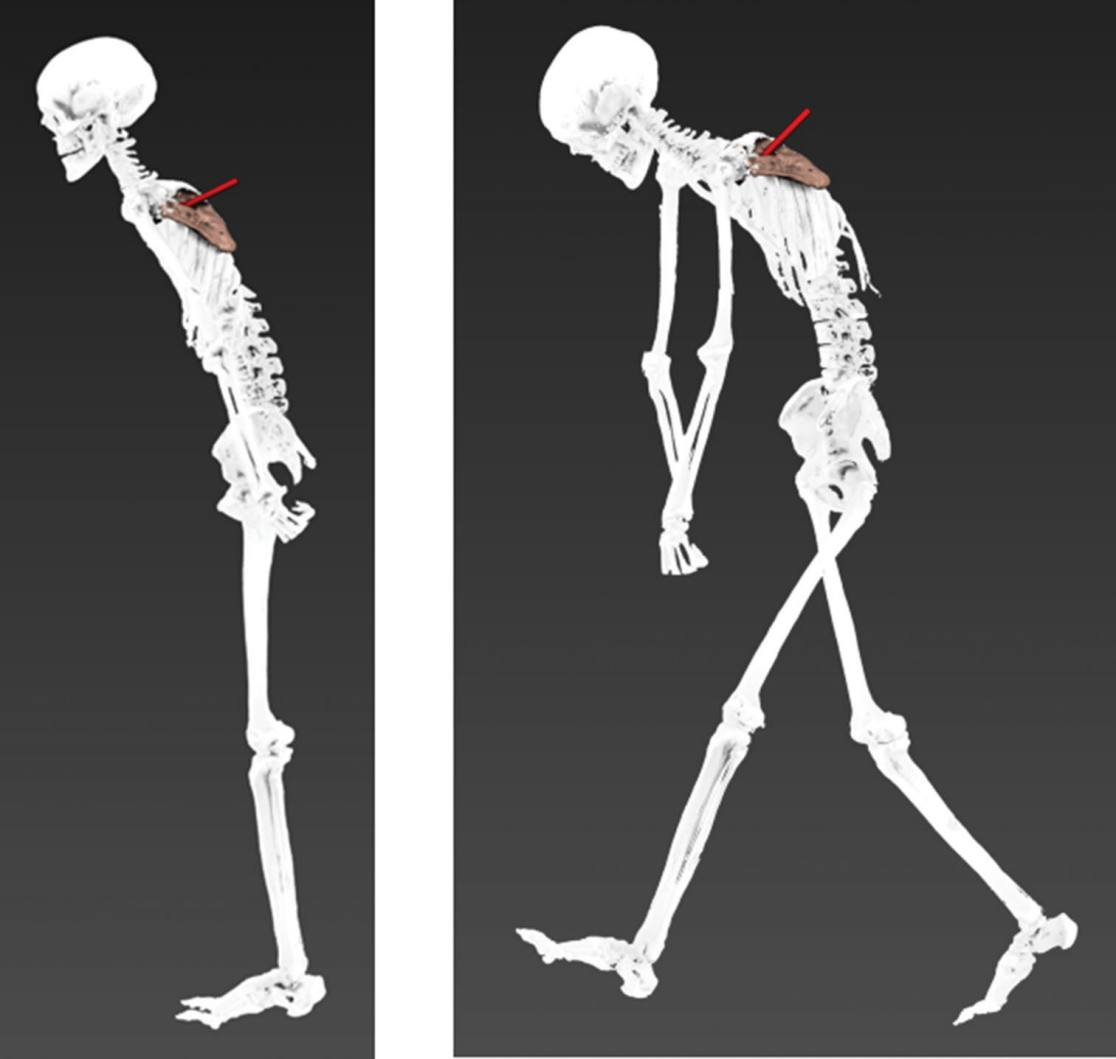
Two possible bending postures of Iceman at the time of the shooting

We then animated Iceman’s entire body, positioning him in an upright standing posture (Fig. 8). This perspective suggests that a forward-facing stance aligns with the trajectory of the arrow at the moment of impact. Subsequently, we simulated various bending positions (Fig. 9), and from the reconstructions, it can be observed that the trajectory is directed downward, indicating that the “perpetrator” was positioned at a higher elevation than Iceman at the time the arrow was fired.

## Discussion 

Determining the cause and manner of death in forensic cases is critical, as understanding how and when a person dies can have significant consequences for crime scene investigations. Forensic pathologists must be accurate and precise, employing all available methods and approaches to address key questions.

In cold or archaeological cases, this task becomes particularly challenging. The body may have undergone post-mortem alterations, and contextual information about the “crime scene” is often unavailable. As demonstrated by the numerous publications on the Iceman case, interpretations of imaging results can lead to differing conclusions, underscoring the importance of periodically reviewing imaging data. This need is amplified as imaging quality improves and advanced post-processing software enables new avenues for analysis.

Although X-rays and CT scanning were performed soon after the Iceman’s discovery in 1991, only in 2001 the arrowhead was identified [30]. It was not until 2003 that both the cause and manner of death were first published [31], attributing the cause of death to bleeding from a puncture wound caused by an arrow and suggesting the manner of death was either accidental or homicidal. In 2007, Perner et al. [32] identified a laceration on the dorsal wall of the left subclavian artery accompanied by an aneurysm. However, a more recent study [33] comparing Iceman’s injuries with clinical data challenges this conclusion, suggesting that he may have survived for several hours after the injury. This finding highlights the importance of cautious interpretation, as the implications differ significantly depending on whether Iceman died immediately or walked for hours, potentially distancing himself from the crime scene.

While we were unable to definitively resolve this question, we objectively quantified the volume of blood loss. The amount was limited to approximately 110 mL, based on 3D reconstruction volume calculations. This volume does not support the theory of acute death due to massive internal bleeding. However, we cannot exclude the possibility of external bleeding contributing to blood loss. In complex cases such as the Iceman’s, where determining the cause of death remains challenging, it is essential to consider alternative hypotheses, including the possibility of hypothermia as a contributing factor.

Animation techniques have been increasingly applied in forensic cases, enabling the reconstruction of probable postures at the time of an incident and providing intuitive visualizations of injury scenarios [2, 3]. By using victim-specific 3D models that accurately represent individual proportions and the exact location of injuries, it is possible to achieve precise and reliable reconstructions [6]. In this study, we were able to clearly visualize that a standing position could account for the observed injury pattern. Whether Iceman was aware of his aggressor or fleeing when struck cannot be determined. However, our findings suggest that his shoulder and arm were in a relaxed position and aligned along the body. A kneeling or semi-upright position, as proposed by Pertner et al. [41], could also explain the trajectory. Additionally, a bent posture might be plausible, but only if the perpetrator was positioned at a higher elevation than Iceman.

This animation technique is still relatively novel, and as Villa et al. [6] emphasize, it presents numerous challenges, particularly in forensic contexts. In archaeological cases, where the dynamics of events are often less constrained by legal procedural standards, the application might seem more straightforward. However, it is crucial to transparently address potential errors and biases in both scenarios. In our analysis, the shoulder and arm were positioned along the side, and the soft tissues were “rigged” to align with the underlying skeletal structure. However, soft tissue movement is inherently complex, and the position of the arrowhead may have been altered post-injury. Animation is notably easier to perform when a definitive “hard” endpoint—such as a fixed skeletal position or impact site—can be established. As demonstrated in our study, even minor adjustments to skeletal positioning can significantly alter angles and trajectories, highlighting the sensitivity of this technique to small changes [42].

## Conclusions

In this study, we have demonstrated the efficacy of 3D segmentation and modeling techniques in analyzing complex forensic injuries in a “cold case”. Through manual 3D segmentation and visualization of Iceman’s shoulder injury, we identified a previously unrecognized area of hematoma. The approximate volume of 110 mL of hematoma was insufficient to account for death by bleeding alone, although we cannot exclude the possibility of external blood loss. Furthermore, by applying animation techniques, we were able to clearly visualize how a straight trajectory aligns the lesions on the scapula with the laceration in the artery, offering additional insight into the nature and mechanism of the injury. These findings underscore the value of advanced imaging and animation methods in forensic pathology, both in contemporary and archaeological cases. They enable more accurate reconstructions of traumatic events and deepen our understanding of the circumstances surrounding the injury.

## Electronic supplementary material

Below is the link to the electronic supplementary material.

Supplementary Material 1

Supplementary Material 2

Supplementary Material 3

Supplementary Material 4

Supplementary Material 5
