# Supplementary figures and images for: Ötzi the Iceman: forensic 3D reconstructions of a 5300-year-ago murder case
Source: Int J Legal Med. 2025 May 21;139(5):2263–71. doi: 10.1007/s00414-025-03510-5 (PMC12354122; doi:10.1007/s00414-025-03510-5)

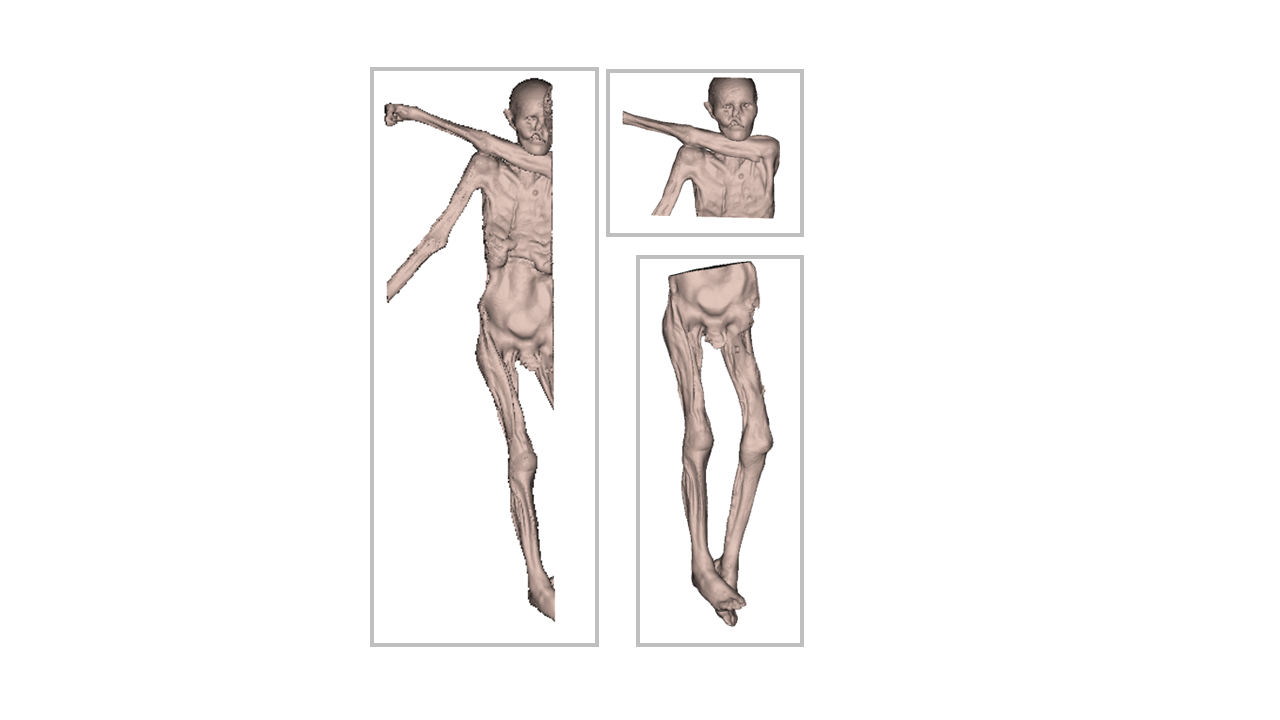

Supplement: Supplementary file 1 — Supplementary Material 1 [file 414_2025_3510_MOESM1_ESM.tif]

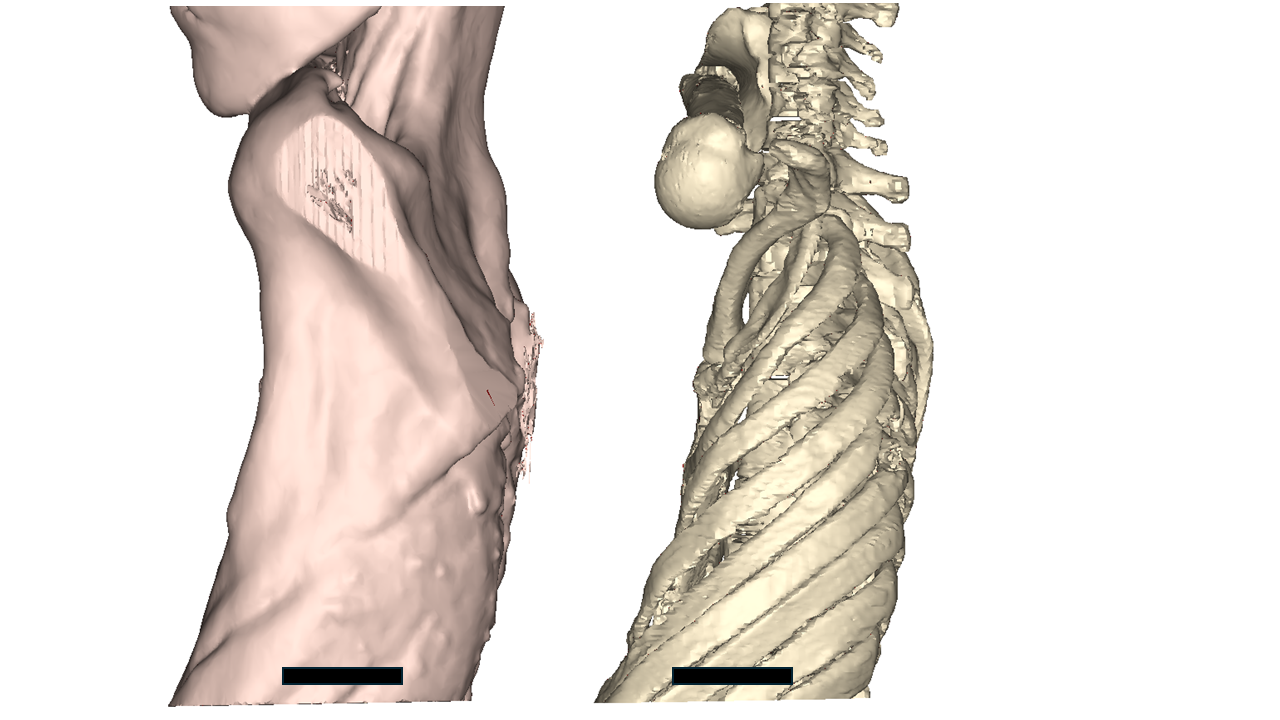

Supplement: Supplementary file 2 — Supplementary Material 2 [file 414_2025_3510_MOESM2_ESM.tif]

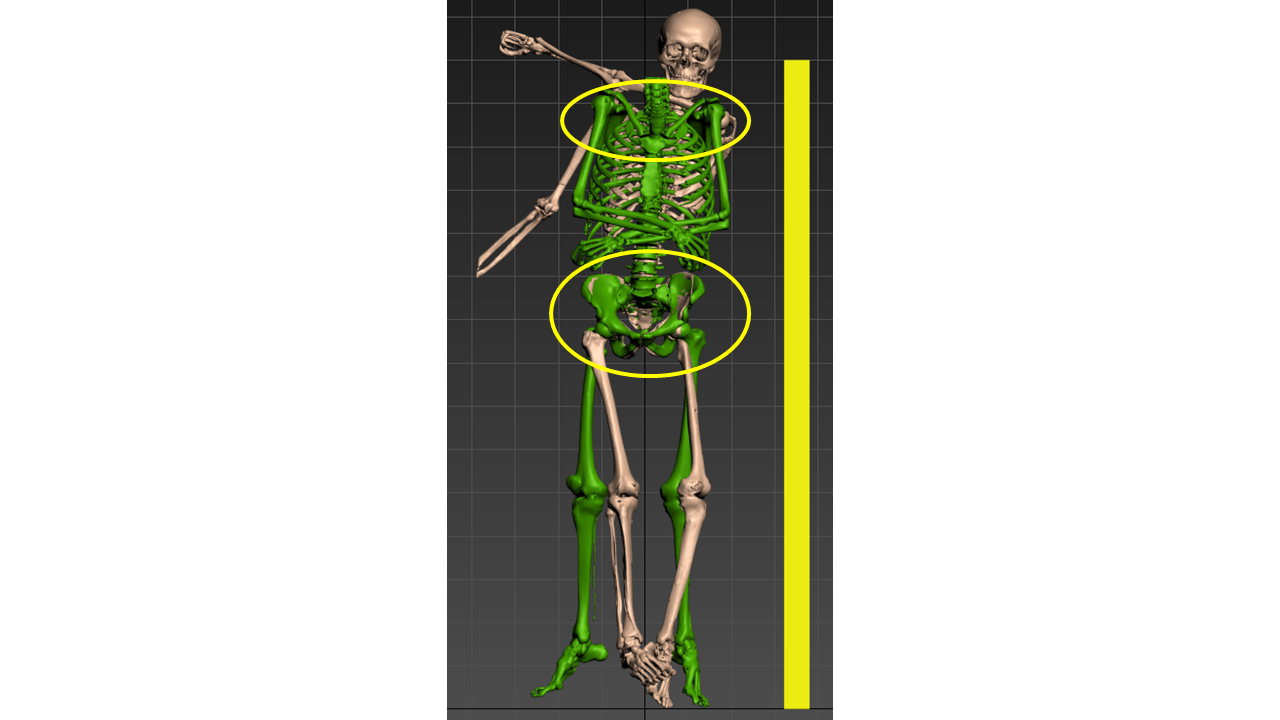

Supplement: Supplementary file 3 — Supplementary Material 3 [file 414_2025_3510_MOESM3_ESM.tif]

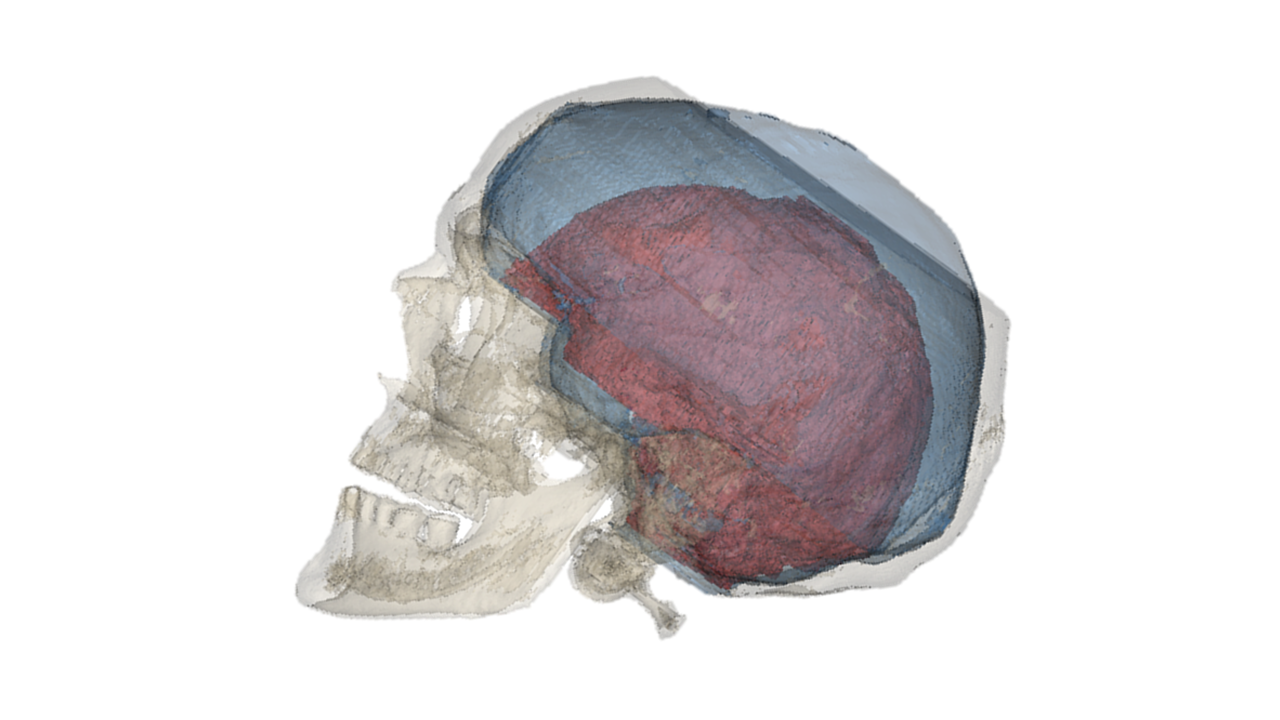

Supplement: Supplementary file 4 — Supplementary Material 4 [file 414_2025_3510_MOESM4_ESM.tif]
