# Supplementary material for: Ötzi the Iceman: forensic 3D reconstructions of a 5300-year-ago murder case
Source: Int J Legal Med. 2025 May 21;139(5):2263–71. doi: 10.1007/s00414-025-03510-5 (PMC12354122; doi:10.1007/s00414-025-03510-5)
Supplement: Supplementary file 5 — Supplementary Material 5 [file 414_2025_3510_MOESM5_ESM.docx]

**Author contributions:**

CV, Conceptualization, Methodology, Formal analysis, Visualization, Writing – original draft, Writing – review and editing

SL Validation, Writing – review and editing

AZ Validation, Writing – review and editing

NL Conceptualization, Validation, Writing – review and editing
